# Relationship between insecure attachment and physical symptom severity is mediated by sensory sensitivity

**DOI:** 10.1002/brb3.1717

**Published:** 2020-06-26

**Authors:** Thao Lan Le, Rose Geist, Jon Hunter, Robert G. Maunder

**Affiliations:** ^1^ Department of Psychiatry Mount Sinai Hospital Toronto ON Canada; ^2^ Department of Psychiatry The Hospital for Sick Children Toronto ON Canada

**Keywords:** attachment, physical symptoms, sensitivity

## Abstract

**Objective:**

Various models have been used to explain somatization, including attachment theory, which describes how formative experiences influence perceptions of vulnerability and threat. Although attachment insecurity is associated with greater physical symptoms, the mechanisms by which attachment insecurity influences the experience of physical symptoms are not clear. Sensory processing sensitivity (SPS) describes a low threshold to responding to stimuli and high emotional reactivity. It is associated with both attachment insecurity and physical symptoms. The purpose of this study is to test a model in which attachment insecurity, depression, and SPS interact to influence physical symptoms.

**Methods:**

Cross‐sectional data from the online Self‐Assessment Kiosk were used (*N* = 186). Participants were surveyed regarding attachment insecurity (ECR‐M16), physical symptom severity (PHQ‐15), sensory processing sensitivity (HSPS), and depression (PHQ‐9). A path analysis was used to analyze the data.

**Results:**

Modal participants were white (74%) single (45%) women (80%) with university education (79%). Attachment anxiety, attachment avoidance, and sensitivity were correlated with physical symptom severity. The data suggested that sensitivity mediates between attachment anxiety and physical symptoms (*β*_indirect_ = 0.070, *p* = .003 and *β*_direct_ = −0.030, *p*> .05) and this relationship remains significant when controlling for depression.

**Conclusions:**

This study extends our understanding of the potential pathways that lead individuals with attachment insecurity to experience burdensome physical symptoms by supporting a mediating role for SPS.

## Graphical abstract

Sensory sensitivity mediates between attachment anxiety and physical symptoms. Findings explain one pathway in which people with attachment insecurity report higher symptom severity.

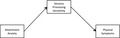

## 1 INTRODUCTION

People who experience multiple physical symptoms have poorer health‐related quality of life (Jackson et al., 2006) and higher healthcare utilization (Barsky, Orav, & Bates, 2005). Furthermore, multiple symptoms increase healthcare use and disability, whether or not symptoms are explained by diseases (Creed et al., 2012; Escobar et al., 2010). Since experiencing multiple symptoms is common (Fink, Sørensen, Engberg, Holm, & Munk‐Jørgensen, 1999) and burdensome, it is important to identify factors that influence the experience. We focus on two: insecure attachment and sensory processing sensitivity (SPS).

Adult attachment theory describes attitudes and behaviors in close relationships that originate in early development (Sibley, 2005). Insecure attachment is often measured along two independent dimensions. Attachment anxiety manifests as concern about rejection and magnified expression of distress (Griffin & Bartholomew, 1994). Attachment avoidance manifests as emotional distance and suppressed expression of distress. Higher insecure attachment, especially attachment anxiety, is associated with more physical symptoms (Grimen & Diseth, 2016; Schroeter et al., 2015; Taylor, Mann, White, & Goldberg, 2000). This has been attributed to a low threshold to appraising physical sensations as a problems and to amplified help‐seeking (Ciechanowski, 2002).

SPS is conceptualized as a trait involving degrees of responsive to environmental stimuli (Aron & Aron, 1997). SPS is correlated with perceived stress, perceived poor health, and greater physical symptom severity (Benham, 2006; Grimen & Diseth, 2016). In addition, each dimension of insecure attachment is associated with SPS (Gülbin, Fulya, & Nebi, 2018; Jerome & Liss, 2005; Meyer & Carver, 2000).

While both attachment insecurity and SPS are conceptualized as stable traits, it is not known which emerges earlier in development, or whether SPS is modifiable by environmental factors, as attachment insecurity is (Waters, Merrick, Treboux, Crowell, & Albersheim, 2000). From its first description, SPS has been associated both with aspects of temperament and early environment (Aron & Aron, 1997). It is possible that SPS may develop or change as a child adapts response strategies to his or her environment. For instance, adopting a highly responsive strategy, involving stronger emotional reactions and complex processing strategies, might be most highly reinforced in environments in which its high energy consumption is warranted.

The purpose of this study is to test a model in which attachment insecurity and SPS interact to influence physical symptom severity. Depressive symptoms are included because of their known associations with each of these variables (Bifulco, Moran, Ball, & Bernazzani, 2002; Engel‐Yeger & Dunn, 2011; Jinyao et al., 2012; Kroenke & Spitzer, 1998; Liss, Timmel, Baxley, & Killingsworth, 2005). We test a hypothesized causal pathway in which attachment insecurity develops prior to SPS and modifies SPS by amplifying signals of potential harm. Higher SPS, when applied to bodily sensations, would then lead to experiencing more severe physical symptoms.

## 2 METHODS

We analyzed data provided by anonymous users of the Self‐Assessment Kiosk (Maunder & Hunter, 2018), a free online resource used by individuals interested in feedback about health‐related constructs. Users select from over 20 validated measures and have the option to consent to research. Research using data from consenting users of the Self‐Assessment Kiosk has been approved by the Mount Sinai Research Ethics Board.

Of 1,711 unique first‐time users between September 2016 and September 2019, data were excluded for those who did not consent (*N* = 394) and those who consented but did not complete all four measures of interest (*N* = 1,131). This left 186 participants.

### 2.1 Measures

Attachment insecurity was measured with a 16‐item modification of the Experiences in Close Relationships‐Revised (Sibley, 2005), the ECR‐M16 (Lo et al., 2009), which is validated for adults with physical illness. Attachment anxiety and attachment avoidance are scored on continuous scales with good internal consistency and test–retest stability.

SPS was measured with the 27‐item Highly Sensitive Persons Scale, which assesses attention to subtleties, being easily overwhelmed by stimuli, and conscientiousness on a continuous unidimensional scale with adequate reliability and content, convergent, and discriminant validity (Aron & Aron, 1997).

Physical symptom burden was measured with the Patient Health Questionnaire (PHQ‐15) which measures how much 15 physical symptoms have bothered the respondent during the past week (Spitzer, 1999). The questionnaire has been found to be adequately reliable. A PHQ‐15 score of three or more had a sensitivity of 78% and specificity of 71% in identifying severe somatic symptoms (van Ravesteijn et al., 2009).

Depressive symptoms were measured with the PHQ‐9. As a screening instrument, a PHQ‐9 score of 10 or greater has a sensitivity of 88% and a specificity of 88% for a major depression (Kroenke, Spitzer, & Williams, 2001).

### 2.2 Analysis

Descriptive statistics and Spearman's rank correlations between SPS, attachment anxiety, attachment avoidance, depression, and physical symptoms were calculated. Path analysis using structural equation modeling (AMOS v.26, IBM, 2019) was used to test the fit of a hypothesized model in which attachment anxiety, attachment avoidance, and depression interact with SPS to influence physical symptoms. The results of path analysis provide estimates of the magnitude and significance of the hypothesized relationships between variables in the path diagram. The fit indices used included the goodness of fit (GFI), normed fit index (NFI), comparative fit indices (CFI), and root mean square error of approximation (RMSEA) (Akaike, 1998). RMSEA values of less than 0.05 (MacCallum, Browne, & Sugawara, 1996) and GFI, NFI, and CFI values of greater than 0.90 (Hu & Bentler, 1999) indicate good fit. The chi‐square test goodness of fit test was also reported as a conventional, commonly reported measure of absolute fit in the literature. Since the chi‐square is highly dependent on sample size, the relative chi‐square (CMIN/DF) was used as a measure of model fit. A value of less than 3 represents acceptable fit (Kline, 1998).

## 3 RESULTS

Consenting first‐time Self‐Assessment Kiosk users who completed the measures of interest differed from those who did not with respect to age (39.6 ± 15.3 years vs. 45.3 ± 15.2 years respectively, *p* < .001), but did not differ by gender, marital status, or the prevalence of at least one reported medical condition (data not shown). Table 1 describes participant characteristics (*N* = 189). The modal participant was a woman (80%), with a graduate degree (45%), who was white (74%) and single (45%). Attachment insecurity, depression, SPS, and physical symptoms were significantly intercorrelated (Table 2).

**TABLE 1:** Characteristics of participants

	*N* = 186	%
Age, mean (*SD*)	39.6 (15.3)	
Men	29	17.7
Women	131	79.9
Education		
Up to high school degree	10	6.1
Any postsecondary degree	23	14.0
Bachelor degree	57	34.8
Graduate or professional degree	73	44.5
Prefer not to answer	1	0.06
Ethnicity		
White	120	74.1
Black	6	3.7
Asian	13	8.0
Other	21	12.9
Prefer not to answer	2	1.2
Marital status		
Single	73	44.8
Separated, divorced or widowed	22	13.5
Married or common law	65	34.9
Prefer not to answer	3	1.8
	***M* (*SD*)**	
Attachment anxiety	4.1 (1.3)	
Attachment avoidance	3.3 (1.3)	
Sensitivity	47.1 (15.6)	
Physical symptoms	7.9 (5.0)	
Depressive symptoms	7.8 (5.8)	

**TABLE 2:** Correlations between variables

	Attachment anxiety	Attachment avoidance	Sensitivity	Physical symptoms
Attachment Anxiety				
Attachment Avoidance	.311*			
Sensitivity	.487*	.259*		
Physical symptoms	.293*	.258*	.478*	
Depression	.391*	.360*	.540*	.622*

* Significant relationships *p *< .05, *N* = 186.

**FIGURE 1:**
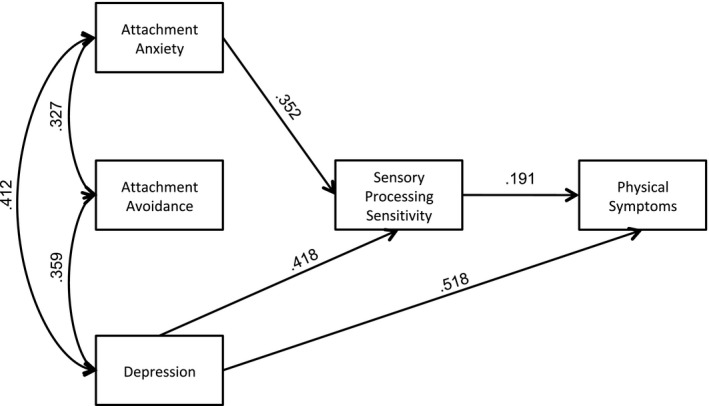
Final model

The results of path analysis with the standardized regression coefficients the relationship of model variables with physical symptoms are presented in Figure 1. This model had a good fit with a chi‐square = 0.544 (*df* = 3, *p* = .91), RMSEA = 0.000, GFI = 0.999, NFI = 0.998, and CFI = 1.000. Figure 1 indicates that attachment anxiety has a significant indirect effect on physical symptoms with SPS as the mediating variable (*β*_indirect_ = 0.070, *p* = .003 and *β_direct_* = −0.030, *p *> .05). Depression has a significant direct and indirect effect on physical symptoms with SPS as the mediating variable (*β*_direct_ = 0.512, *p* = 0.001 and *β*_indirect_ = 0, *p* = .004). However, attachment avoidance has no significant effect on symptom severity (*β*_indirect_ = 0.030, *p* = .676 and *β*_direct_ = 0.037, *p* = .541).

## 4 DISCUSSION

These results are consistent with a model in which attachment anxiety develops before SPS, influences the development of SPS, and influences symptom severity indirectly through SPS as a mediator of this effect. Thus, we extend current understanding of mechanisms by which attachment anxiety amplifies physical symptoms.

Prior work documents a correlation between attachment anxiety and reporting physical symptoms (Ciechanowski, 2002). We find that this relationship is fully mediated by SPS. This is consistent with the hypothesis that in early development, elevated attachment anxiety influences sensitivity to both internal and external cues (reinforcing SPS), which in turn leads to appraising changes in internal sensations as potentially harmful, and thus to being more bothered by physical symptoms. This meditating relationship was significant even after taking depressive symptoms into account.

Limitations of the study include its use of data collected through an internet self‐assessment resource, leading to a bias of self‐selection favoring participants who are concerned about their health. Other limits on generalizability are that the majority of participants were educated white women. Women tend to score higher on the HSP scale (Aron & Aron, 1997). Of note, participants from this cohort reported higher attachment anxiety and avoidance than those recruited from a family medicine clinic (Le, Levitan, Mann, & Maunder, 2018), which is consistent with biases related to self‐selection. Although physical symptom severity and SPS always rely on self‐report, observer‐rated measures of adult attachment are available, but were not used. While we provide evidence that the model in which SPS mediates between attachment anxiety and physical symptoms is plausible, other causal relationships are also possible. For instance, experiencing severe symptom could amplify both attachment insecurity and sensitivity to stimuli. A cross‐sectional study cannot distinguish causal paths, but indicates that longitudinal research justified.

## 5 CONCLUSION

This study extends our understanding of the potential pathways that lead individuals with attachment insecurity to experience burdensome physical symptoms by supporting a mediating role for SPS.

## CONFLICTS OF INTEREST

None declared.

## AUTHOR CONTRIBUTION

All authors conceived the study. TL and RM performed the analyses. TL conducted the literature search and wrote the first draft of the manuscript. All authors contributed to and have approved the final manuscript.

## Data Availability

The data that support the findings of this study are available from the corresponding author upon reasonable request.
